# Supplementary material for: Dietary Habits and Tusk Usage of Shovel-Tusked Gomphotheres from Florida: Evidence from Stereoscopic Wear of Molars and Upper and Lower Tusks
Source: Biology (Basel). 2022 Nov 30;11(12):1748. doi: 10.3390/biology11121748 (PMC9774678; doi:10.3390/biology11121748)
Supplement: Supplementary file 1 [file biology-11-01748-s001.zip › Supplementary Data Table S2.pdf]

Table S2. ANOVA and Tukey's HSD test results for differences in numbers of pits, numbers of scratches, and numbers of large pits, Significant differences are indicated in bold.

**Number of Pits**						
**ANOVA Results – Test for Equal Means**						
	**Sum of Squares**	**df**	**Mean square**	**F**	***p***	
Between groups:	4649.55	5	929.909	32.95	5.167E-20	
Within groups:	2934,83	104	28.2195			
Total:	7584.37	109	1E-05			
**Tukey's honest significance test: Pair-wise comparisons - q values below the diagonal, p values above the diagonal.**						
	Amebelodon floridanus	*Serbelodon barbourensis*	*Konobelodon britti*	*Loxodonta cyclotis*	*Loxodonta africana*	*Elephas maximus*
Amebelodon floridanus		**4.188E-05**	**7.69E-05**	0.06055	**0**	**4.67E-12**
Serbelodon barbourensis	6.999	-	0.4428	0.9575	0.1223	0.0556
Konobelodon britti	6.788	2.607	-	0.2006	0.9951	1
Loxodonta cyclotis	4.002	1.2	3.263	-	**0.04269**	**0.01853**
Loxodonta africana	15.83	3.592	0.7425	4.19	-	0.9116
Elephas maximus	11.81	4.049	0.1078	4.605	1.437	-
**Number of Scratches**						
**ANOVA Results – Test for Equal Means**						
	**Sum of Squares**	**df**	**Mean square**	**F**	***p***	
Between groups:	1317.51	5	263.503	12.61	1.413E-09	
Within groups:	2173.55	104	20.8995			
Total:	3491.07	109				
**Tukey's honest significance test: Pair-wise comparisons - q values below the diagonal, p values above the diagonal.**						
	Amebelodon floridanus	*Serbelodon barbourensis*	*Konobelodon britti*	*Loxodonta cyclotis*	*Loxodonta africana*	*Elephas maximus*
Amebelodon floridanus	-	0.5902	1	0.7194	**3.22E-09**	**1.891E-05**

Serbelodon barbourensis	2.285	-	0.9738	1	**0.03486**	0.05632
Konobelodon britti	0.1124	1.072	-	0.9671	0.1135	0.09523
Loxodonta cyclotis	1.998	0.1986	1.13	-	0.2368	0.2115
Loxodonta africana	9.953	4.295	3.638	3.142	-	0.9948
Elephas maximus	7.269	4.042	3.744	3.225	0.7505	-
**Number of Large Pits**						
**ANOVA Results – Test for Equal Means**						
	**Sum of Squares**	**df**	**Mean square**	**F**	***p***	
Between groups:	140.031	5	28.0063	2.609	0.029	
Within groups:	1105.53	103	10.7333			
Total:	1245.56	108	0.02579			
**Tukey's honest significance test: Pair-wise comparisons - q values below the diagonal, p values above the diagonal.**						
	Amebelodon floridanus	*Serbelodon barbourensis*	*Konobelodon britti*	*Loxodonta cyclotis*	*Loxodonta africana*	*Elephas maximus*
Amebelodon floridanus	-	**0.01467**	0.9954	0.9993	0.5824	0.7956
Serbelodon barbourensis	4.716	-	0.2528	0.4054	0.281	0.6414
Konobelodon britti	0.73	3.093	-	0.987	0.8691	0.8618
Loxodonta cyclotis	0.4908	2.693	0.9157	-	0.9961	0.9927
Loxodonta africana	2.302	3.01	1.594	0.7075	-	1
Elephas maximus	1.809	2.173	1.618	0.8081	0.283	-
